# An exploration of the successful scale-up of the electronic community health information system in Kenya

**DOI:** 10.1093/oodh/oqaf020

**Published:** 2025-09-13

**Authors:** Salome Ireri, Peter Waiganjo, Daniel Orwa Ochieng, Michael Kagiri, Michael Anindo, Maureen Adoyo, Rachael Wanjiru, Joan Kirui, Raphael Pundo, Maureen Kimani, John Wanyungu

**Affiliations:** Centre for Health Informatics and Digital Health, Department of Computing and Informatics, Faculty of Science and Technology, University of Nairobi, Chiromo Campus, Riverside Drive, P.O. Box 30197 - 00100, Nairobi City, Nairobi County, Kenya; Centre for Health Informatics and Digital Health, Department of Computing and Informatics, Faculty of Science and Technology, University of Nairobi, Chiromo Campus, Riverside Drive, P.O. Box 30197 - 00100, Nairobi City, Nairobi County, Kenya; HealthIT, University of Nairobi, Main Campus, UoN Towers, 12th floor, P.O. Box 68241 - 00200, Nairobi City, Nairobi County, Kenya; Centre for Health Informatics and Digital Health, Department of Computing and Informatics, Faculty of Science and Technology, University of Nairobi, Chiromo Campus, Riverside Drive, P.O. Box 30197 - 00100, Nairobi City, Nairobi County, Kenya; Centre for Health Informatics and Digital Health, Department of Computing and Informatics, Faculty of Science and Technology, University of Nairobi, Chiromo Campus, Riverside Drive, P.O. Box 30197 - 00100, Nairobi City, Nairobi County, Kenya; Centre for Health Informatics and Digital Health, Department of Computing and Informatics, Faculty of Science and Technology, University of Nairobi, Chiromo Campus, Riverside Drive, P.O. Box 30197 - 00100, Nairobi City, Nairobi County, Kenya; HealthIT, University of Nairobi, Main Campus, UoN Towers, 12th floor, P.O. Box 68241 - 00200, Nairobi City, Nairobi County, Kenya; Department of Health Sciences, Rongo University, Kitere Hill, Kisii - Migori Highway, P.O. Box 103 - 40404, Rongo Town, Migori County, Kenya; HealthIT, University of Nairobi, Main Campus, UoN Towers, 12th floor, P.O. Box 68241 - 00200, Nairobi City, Nairobi County, Kenya; HealthIT, University of Nairobi, Main Campus, UoN Towers, 12th floor, P.O. Box 68241 - 00200, Nairobi City, Nairobi County, Kenya; HealthIT, University of Nairobi, Main Campus, UoN Towers, 12th floor, P.O. Box 68241 - 00200, Nairobi City, Nairobi County, Kenya; Division of Community Health Services, Ministry of Health, Afya House, Cathedral Road, Upper Hill, P.O. Box 30016 - 00100, Nairobi City, Nairobi County, Kenya; Division of Community Health Services, Ministry of Health, Afya House, Cathedral Road, Upper Hill, P.O. Box 30016 - 00100, Nairobi City, Nairobi County, Kenya

**Keywords:** health information system, electronic community health information system, scale-up, approaches, enablers, barriers

## Abstract

Healthcare delivery is swiftly evolving, adopting digital solutions to significantly enhance efficiency and effectiveness. To improve community health service delivery and advance Universal Health Coverage in Kenya, a countrywide Electronic Community Health Information System (eCHIS) was implemented. This study investigated the approaches, enablers and barriers influencing its scale-up from pilot to national level. A qualitative evaluation of the eCHIS scale-up process was conducted, involving key informant interviews with policymakers at the Ministry of Health Kenya, implementing partners and county health teams and focus group discussions with County Health Focal Persons from selected counties at the subnational level. eCHIS has been implemented countrywide. The Ministry of Health at the national level employed a sequential approach, where a pilot informed version two, which was then deployed county by county. Counties at the subnational level, however, had autonomy to select either sequential, deploying eCHIS incrementally in one subcounty at a time, or non-sequential, deploying eCHIS in all subcounties simultaneously, methods tailored to their specific context and factors. Scale-up enablers included strong leadership, supportive policies, adequate financing and resources, partnerships, readiness assessments, stakeholder engagement, contextual adaptation, training, monitoring and evaluation of outputs, infrastructure and interoperability and centralized management. Barriers included funding and resource limitations, logistical and infrastructure challenges, communication challenges and partner capacity shortfalls. This study explored these aspects of scale-up in depth and provides insights for policymakers and implementers navigating the complex landscape of Health Information Systems scale-up. These findings can inform the development of guidelines for future HIS scale-up efforts.

## INTRODUCTION

Deficiencies in equitable access and healthcare quality are prevalent issues within many healthcare systems [1]. Implementing Health Information Systems (HIS) offers a promising avenue for substantially improving healthcare delivery and facilitating Universal Health Coverage (UHC), a critical objective for low- and middle-income countries (LMICs) [2–4].

A HIS is designed to securely and efficiently manage health information and data, thereby enabling evidence-based decision-making [5]. HIS typically integrate diverse functionalities, including clinical support, patient management, administrative and financial operations, data analytics, reporting and mechanisms for health information exchange [6].

The demonstrated potential of HIS to improve healthcare delivery at both small and large scales has led to a significant increase in piloting within LMICs [7]. Pilots cost-effectively assess HIS feasibility and benefits [8], providing essential data and insights for decisions on scaling up HIS to maximize population-level impact [3, 9]. However, a concurrent rise in ‘pilotitis’ which is the tendency to initiate numerous small-scale pilot projects without clear pathways or commitment to full-scale implementation, has also been observed [3].

The scale-up of HIS involves the intentional expansion of proven interventions to serve larger populations sustainably, promoting long-term healthcare system improvements [10]. According to the ExpandNet/WHO scaling-up framework, the scale-up process follows an approach or strategy and involves horizontal expansion, broadening an intervention’s reach to additional areas or populations and vertical integration, embedding the intervention into the healthcare system through policy, regulatory and budgetary changes [11]. Determinants of scale-up comprise scale-up enablers that drive the process and barriers that hinder it [12–15]. Successfully scaling a HIS intervention can inform future efforts and potentially mitigate the common challenge of ‘pilotitis’.

This study sought to understand how the Electronic Community Health Information System (eCHIS) in Kenya was scaled-up from pilot to countrywide implementation. Specifically, the study aimed to explore the approaches used to scale-up eCHIS, to ascertain the key enablers of the scale-up of eCHIS and to identify the barriers that were encountered during the scale-up process.

### Digital health landscape in Kenya

Kenya’s development goals are guided by strategic frameworks reviewed every 4 years to align with the government’s vision of national prosperity. The current ‘Big 4 Agenda’ prioritizes UHC as one of its key pillars and as a strategic initiative towards achieving Sustainable Development Goal – 3 [16, 17]. The government recognizes the transformative potential of Digital Health Technologies such as HIS in healthcare delivery, especially within LMIC contexts [2], and is therefore leveraging these technologies to achieve UHC. Consequently, the government is prioritizing HIS implementations across all 47 counties. These initiatives, detailed in Kenya’s UHC Policy 2020–2030, aim to digitize records, enhance information exchange and facilitate paperless referrals, therefore enhancing healthcare delivery efficiency and effectiveness [18].

HIS implementation in Kenya involves both national and county governments, as Kenya’s 2010 Constitution devolved primary healthcare delivery from the Ministry of Health (MoH) to the 47 subnational governments to improve service access, equity and quality [18, 19]. The devolved healthcare structure mandates shared national-county responsibility for policy, legislation and resource allocation, ensuring cohesion and support for the counties. It also promotes community involvement, enhancing participation and accountability in healthcare decision-making [20].

In the last decade, there has been a rapid transition in Kenya towards the use of HIS to enhance healthcare service delivery [21]. This digital transformation is underpinned by government initiatives such as the Digital Health Authority, which fosters a national digital health ecosystem [22], and the Digital Health Superhighway, enabling interoperable Health Information Exchange (HIE) [23]. Examples of HIS that have implemented include the Kenya Health Information System, KenyaEMR and the eCHIS [21, 24–26].

### Electronic community health information system in Kenya

Recent efforts have intensified to strengthen primary healthcare as a key enabler for UHC. A significant initiative in this regard is the digitization of community health services, addressing challenges inherent in the traditional paper-based record-keeping used by Community Health Workers, now known as Community Health Promoters (CHPs) [27]. In 2020, the Kenyan government initiated the development of a national community-based HIS. eCHIS deployment began with a pilot in Kisumu County in mid-2021, followed by a national rollout commencing in mid-2022, aiming to include all 107 000 Kenyan CHPs by 2025 [28]. The eCHIS implementation was a collaboration between the MoH, county governments and over 25 implementing partners, including Medic Mobile, Living Goods, LWALA, USAID, HealthIT, AMREF, Kenya Red Cross, LVCT Health, World Vision, Intra Health, Palladium Group, Save the Children, JSI inSupply Health, WHO, UNICEF, PATH, Jhpiego, Population Council, JICA and Safaricom [29, 30]. Partner selection criteria were guided by partners’ demonstrated capacity to contribute to eCHIS implementation through support to MoH divisions, departments and county governments. Specifically, partners were chosen for their ability to facilitate policy operationalization, strengthen supply chain systems, deliver targeted capacity-building initiatives, develop and maintain technological solutions, provide essential infrastructure, enhance quality management processes and support monitoring and evaluation activities [30].

Based on the standardized Community Health Toolkit (CHT) framework by Medic Mobile [31], eCHIS seeks to improve healthcare quality, equity and outcomes for Kenyan households by empowering CHPs and enhancing data-driven decision-making for promotive and preventive care [28]. The system is designed for household enrollment, service delivery tracking, supply chain management, community health monitoring and client messaging [32]. Additionally, it supports HIE, enabling data integration with Electronic Medical Records (EMR) systems at community health facilities. This allows for seamless patient referrals by CHPs to these facilities. Its primary users are CHPs and the Community Health Assistants (CHAs) who supervise and support them [32].

## MATERIALS AND METHODS

This exploratory case study examined the approaches, enablers and barriers to scaling up eCHIS in Kenya. To ensure participants possessed relevant expertise within time and cost constraints, purposive sampling targeted individuals directly involved in the eCHIS scale-up. The study population included policymakers from community health and primary healthcare, program managers, health professionals and digital solutions experts, among other stakeholders. A total of 38 participants were purposively selected, drawn from five relevant MoH divisions within the eCHIS implementation team, 12 counties and three implementing partner organizations directly involved in the system’s scale-up. Of these, 32 participants responded, yielding a response rate of 84%.

Data were collected through Key Informant Interviews (KIIs) and Focus Group Discussions (FGDs). The corresponding data collection tools were pretested with selected members of the MoH eCHIS implementation team who were not involved in the main study. This process aimed to assess the tools’ validity, reliability and overall effectiveness, as well as to ensure that questions were appropriately mapped to the correct categories of participants, with guidance from the MoH team. In addition, the research team carried out several dry runs to evaluate the logical flow of the questions and to identify and eliminate any ambiguities in wording.

KIIs were conducted with individuals from the MoH’s Divisions of Community Health, Primary Healthcare, Information and Communication Technology, Health Informatics and Digital Health. Additionally, interviews involved CHAs and CHPs from Kakamega, Kisumu and Migori County Health Teams, alongside implementing partners: Medic Mobile, which provided the CHT framework on which eCHIS was built [31], the Lwala Community Alliance, which piloted version two of eCHIS in Migori County [29] and HealthIT, which was responsible for project monitoring, training and user support [30]. FGDs engaged County Health Focal Persons (CHFPs) from Embu, Bomet, Kakamega, Kirinyaga, Kiambu, Kisumu, Migori, Nairobi, Nakuru, Kilifi, Marsabit and Tana River Counties. KIIs and FGDs were recorded using audio recording devises to ensure that participants’ responses were captured accurately. In cases where recording was not possible, detailed field notes were taken.

The selected counties represented diverse settings at Kenya’s subnational level, encompassing pioneer implementers, including pilot counties (implementing between mid-2021 and mid-2023), mid-phase implementers, where over 30 counties out of the 47 in the Country implemented eCHIS (mid-2023 to mid-2024), and late-phase implementers of the HIS (commencing after mid-2024), thereby providing a comprehensive view of the scale-up process from pilot to national implementation.

Following data collection, the recordings were later transcribed verbatim and qualitative thematic analysis was conducted. Responses from both KIIs and FGDs were manually coded by four members of the research team and organized around three emergent key themes: context and scale-up approaches, enablers (facilitators) of scale-up and scale-up barriers (challenges). These thematic data were subsequently analyzed and interpreted.

This study received administrative clearance from the MoH and relevant county health heads, ethical approval from the Chuka University Institutional Ethics Review Committee, and permission from the National Commission for Science, Technology and Innovation. Informed consent was obtained from all participants after they were briefed on the study’s purpose and that the interviews would be recorded. During data analysis, identifying information was removed and codes were assigned to ensure anonymity.

## RESULTS

The findings of this exploratory study are organized around key themes derived from the analysis, corresponding to the study’s objectives. The distribution of the study participants is as follows.

### Distribution of participants by organization

The distribution of participants by organization was as follows: majority were from the counties health teams (71.9%), followed by the MoH (15.6%) and implementing partners (12.5%).

### Distribution of participants by profession

The participants from these organization comprised of: policy makers (9.375%) who are senior ministry officials, program managers (6.25%), digital health managers (6.25%), digital health solutions developers (6.25%) who are part of the development and deployment teams, CHFPs (40.625%) who manage eCHIS implementation and utilization at the county level, including monitoring reporting via the eCHIS dashboard, CHAs (12.5%) who supervise CHPs, and CHPs (18.75%) who directly use eCHIS to deliver community health services.

### Distribution of participant from counties by implementation phase

The distribution of participants from the county health teams by their eCHIS adoption phase was as follows: pioneer implementers (52.2%), the mid-phase implementers (39.1%) and late-phase implementers (8.7%).

### Choice of pilot county

The study revealed that a landscape assessment was conducted in 2020 across selected counties, including the pilot counties of Kisumu and Migori, in preparation for the eCHIS countrywide rollout [33]. According to the report, the MoH opted to pilot eCHIS in Kisumu because the county already had an operational countywide community-based program, SmartHealth by Living Goods. SmartHealth, an open-source mobile platform based on the CHT framework, featured functionalities similar to eCHIS, such as household enrollment and client referral management. This existing infrastructure and support from Living Goods as an implementation partner were leveraged for the eCHIS pilot.

When asked why Kisumu County was chosen for the pilot, one participant from a partner organization highlighted the importance of partnerships and existing infrastructure:

*‘There were already community-based digital health interventions in place, though they were specific to certain programs. Leaning on the existing infrastructure and working with the intervention implementers simplified the process. The eCHIS 2020 Landscape Assessment helped determine the starting point for implementation. Partnerships and infrastructure were key, and this guided where to go next.’* (KII19).

### eCHIS scale-up approaches

The MoH at the national level employed a phased eCHIS rollout across counties, beginning with a pilot in Kisumu County in 2021. In 2022, version two of eCHIS was introduced in Migori County, followed by a gradual rollout to other counties, with full scale-up expected by 2025 [8]. Currently, eCHIS is implemented in all 47 counties, with late-phase implementers Garissa, Mandera, Tana River and Wajir onboarding in mid-2024. Nationwide, eCHIS utilization extends to 95% of the ~107 000 CHPs.

Different scale-up approaches were used in each county. The MoH did not mandate a single approach, allowing counties the flexibility to select methods best aligned with their capacities and local contexts, considering unique enablers, barriers and stakeholder engagement. These strategies included a sequential approach, introducing eCHIS one subcounty at a time, adopted by 35 of the 47 counties including Embu, Kisumu and Migori, and a non-sequential approach, simultaneous implementation across all subcounties, used in counties such as Kakamega and Nakuru.

Kisumu, the pilot county, employed a sequential approach, starting eCHIS implementation in two subcounties in August 2021 and achieving full operation across all seven subcounties by March 2022. Nakuru County, conversely, adopted a non-sequential approach, implementing eCHIS in all its subcounties at once. Nakuru distinguished itself in the implementation of eCHIS by acting as a pioneer implementer, launching the system immediately following the pilot phase. Notably, it was the first county to achieve a successful non-sequential scale-up across the entire county as one participant explained:

*‘Nakuru County had one of the most successful eCHIS implementations,… it onboarded all its11 subcounties at one go and was the first to do so… The process started once eCHIS version two pilot was completed in Migori…’* (FGD2–4).

These approaches were not based on a defined scale-up framework, such as the ExpandNet/WHO Scaling-up framework [11]. Although the use of a scale-up framework was mentioned during planning [28], none was ultimately employed. Instead, eCHIS scale-up was primarily guided by the national community health digitization strategy 2020–2025 [33] and county-specific contextual factors, including resource availability, implementing partner presence and subnational government support. These factors correspond closely with enabler and barriers described in existing digital health intervention scale-up frameworks, such as leadership, stakeholder engagement and capacity building as enablers, and resource limitations and infrastructure gaps as barriers, in the ExpandNet/WHO Scaling-up framework [11]. In response to questions about a guiding scale-up framework, a ministry official indicated:

*‘Regarding the scaling of eCHIS from pilot projects to broader implementation, there was no formal structured framework. The process was more organic and adaptive rather than rigidly planned...’* (KII4).

### Enablers of eCHIS scale-up

During the KIIs and FGDs, participants highlighted 11 important factors as enablers of eCHIS scale-up. These include: Strong leadership and government support, supportive policies and regulations, adequate financing and resources, collaborative efforts and partnerships, contextual adaptation, county readiness assessments, stakeholder engagement, training and capacity building, monitoring and evaluation of outputs, infrastructure and interoperability and centralized management.

#### Strong leadership and government support

Strong leadership emerged as a key enabler from the data. The critical importance of support and clear strategic direction from both national and county government levels played a pivotal role in the eCHIS scale-up. Participants consistently emphasized the government’s key role. At the national level, its endorsement and overarching vision were highlighted as foundational. As one senior ministry official articulated:

*‘The rapid scale-up of eCHIS to 41 counties within seven months was driven by multiple factors. First, there was strong political will, as eCHIS was part of the presidential agenda, creating top-down pressure for swift implementation. … The government procured approximately 110,000 smartphones distributed nationwide, which were essential for the community-level digitization.’* (KII4).

Furthermore, county-level facilitation often entailed strategic collaborations with implementing partners who were vital for mobilizing necessary resources. For instance, a CHFP noted:

*‘The counties couldn’t do it all alone. Our governor supported our implementing partner. … The partner organization came in with crucial financial support, and just as importantly, they provided resources for things like training our users on the new system.’* (FGD1–3).

#### Policies and regulations

The establishment of appropriate policies and regulations was recognized as fundamental to the implementation of eCHIS. The MoH ensures that guiding policies and regulations precede the development and implementation of systems in Kenya. Key frameworks include national policies for HIS, mHealth and community health, alongside the Digital Health and Data Protection Acts. A senior ministry official explained:

*‘At the onset of eCHIS there was a community health policy / community health strategy 2025 to 2030. There is also the Community health framework that outlines community health delivery in the country.’* (KII2).

An implementing partner further articulated the importance of a conducive policy environment for advancing healthcare initiatives:

*‘Regarding the influence of regulations and policies on the implementation of HISs and Smart Primary Care Networks (PCNs) there was support from government objectives and policies in driving the implementation process. ... These policies have played a significant role in ensuring the success of healthcare technology initiatives in Kenya.’* (KII18).

#### Adequate financing and resources

The availability of adequate finances and resources as a crucial factor in the scale-up of eCHIS. eCHIS implementation and scale-up was financed through a composite model, incorporating contributions from both government budgets and support from partner organizations. Counties that achieved simultaneous eCHIS implementation across all subcounties evidently possessed the requisite financial and material resources for concurrent training and deployment. This capacity was particularly demonstrated in counties with support from partner organizations, highlighting a positive correlation between such partnerships and enhanced resource availability. A CHFP elaborated on the nature of this support:

*‘Partnerships were essential for us. They helped bridge the financial gaps and also brought in expertise for capacity building, especially the initial training of our CHAs and CHPs on the eCHIS platform.’* (FGD2–4).

The national and county governments facilitate the provision of stipends to CHPs who formerly served as uncompensated volunteers. This previous lack of remuneration contributed significantly to high attrition rates and compromised the reliability of monthly data collection and reporting. The introduction of these stipends aimed to enhance CHP motivation and retention. A CHP expressed their motivation about using eCHIS, as follows:

*‘Receiving the stipend is a big encouragement, as it shows that our contributions are recognized. …. eCHIS has also transformed my work. Previously, the manual paperwork for reporting was time-consuming ... now, with eCHIS, I can input data in a very short time … this is very motivating.’* (KII12).

#### Collaborative efforts and partnerships

The scale-up to all counties in the country was guided by criteria developed collaboratively by the MoH, county health teams and implementation partners. The MoH engaged over 25 organizations for the eCHIS implementation, bringing in additional expertise and resources. This collaborative approach meant that MoH relied on support from these partners for system development, training and infrastructure. Positive feedback from partners also indicated the project’s potential for successful scale-up. An interview with a ministry official underscored the importance of collaboration with partners to achieve a countrywide scale-up of eCHIS:

*‘It is a significant achievement to have rolled out eCHIS to 41 out of 47 counties within six to seven months, a process initially expected to take three to five years. This was achieved through collaborations with partners that provided the resources that were needed.’* (KII4).

Further insights into the implementation process and the available support were offered by a participant from a partner organization:

*‘With collaboration between the MoH and partners covering the initial 10 counties. … The process involved significant resources for device acquisition, development, training, and implementation. … The collaborative efforts involved in eCHIS implementation, at first involved defining the vision and translating service elements into software. … There were challenges in standardizing data collection processes, but partners provided expertise and resources, including software development engineers to bridge gaps in the design and development process.’* (KII19).

#### Stakeholder engagement

During the pilot phase, user feedback sessions with CHPs and CHAs were instrumental for stakeholder engagement, fostering a deeper understanding of the operational landscape and local contexts, including user literacy levels. A ministry official explained:

*‘There was concern that some of the CHPs could not use smartphones and understand the eCHIS but contrary to this, through observation and feedback sessions, we established that majority knew how to use a smartphone and understood English and therefore could use eCHIS during the pilot in Kisumu. This in part encouraged rollout to other counties. … In Kajiado where we thought that literacy might be a problem, they are actually very grateful for the new system.’* (KII3).

Stakeholder engagement also served as a vital mechanism for identifying and effectively resolving emergent system challenges. A CHFP noted:

*‘Then we also had scheduled meetings whereby, for example, a while back we had all the 47 CHPs brought together to have discussions surrounding eCHIS and the implementation and how far we are at, and challenges that emerged were addressed.’* (FGD1–1).

#### Contextual adaptation

Effective eCHIS scale-up in Kenya is significantly enabled by contextual adaptation. The country’s devolved healthcare system makes it crucial for the MoH to apply flexible approaches when implementing digital health solutions and other health interventions. This is because of the unique local contexts at the subnational governance level, which require careful consideration to facilitate widespread scale-up of interventions. As a CHFP official articulated:

*‘A one-size-fits-all approach to scale-up could not be used. … some counties had the necessary resources to do a full-blown scale-up while others did not. … There are counties that are more mature than others when it comes to the use of digital health systems, … they have implementation guidelines in place.’* (FDG1–6).

#### County readiness assessments

County readiness assessments were critical and were conducted as part of the eCHIS 2020 Landscape Assessment. This ensured that only counties, such as Kisumu, possessing the necessary infrastructure and support mechanisms, such as implementing partners, were shortlisted for the pilot phase, thereby mitigating the risk of failure. Furthermore, considerable emphasis was placed on stakeholder engagement, encompassing system users and partner organizations. As a participant from a partner organization articulated:

*‘Scale-up in itself or just a thought about scale up in itself, was a scary thought. Because before even the government came and made the investment in it, it felt very unpromising. One thing that the ministry had done was, with support from partners, to come up with evaluation criteria to determine county readiness levels. From the point of view of the existing functional community-based systems, and the supporting partner, then to engage for them in adopting the system.’* (KII16).

Additionally, a CHFP noted that insights from the readiness assessment were crucial in averting failures during county rollouts:

*“Before eCHIS was introduced, some counties had already been working with partners on similar projects, facilitating a smoother transition. Although there were minor challenges at the beginning of rollout, they did not hinder the overall acceptance and rollout. … Our experience was important and this was established in the 2020 [landscape] assessment.* (FGD2–1).

#### Training and capacity building

This was a critical element for eCHIS scale-up to occur. Proper training of trainers ensured that the uptake of the eCHIS by the users was high and positive. Ensuring that participants were able to navigate the training and understand the material during the first training ensured that the pilot was successful in terms of participant engagement and comprehension. Overcoming fear and ensuring that participants felt confident and capable was also an essential aspect of ensuring eCHIS acceptance and scale-up. A senior ministry official explained:

*‘There was a well-defined capacity building structure where we had the training of the ministry personnel and eventually we brought on board the Trainers of Trainers from the various counties and then … down to the CHPs. … Having a structure ensured everyone was trained and increased acceptance or eCHIS.’* (KII3).

A CHFP further explained the importance of well-executed training, emphasizing its contribution to user competence:

*‘Once the phones arrived, our partner did a great job at the beginning, ensuring that all CHPs and CHAs understood eCHIS and felt confident using it. … The CHAs could help the CHPs with challenges they encountered once in the field.’* (KII10).

#### Monitoring and evaluation of outputs

Dashboards and indicators were highlighted as crucial tools for monitoring and evaluating the effectiveness of scale-up. They enabled the tracking of diverse metrics to verify operational integrity, with success contingent upon achieving specific benchmarks, including the number of registered CHPs and households. Dashboard reports have made work easier as indicated by a CHFP:

*‘The use of dashboards to monitor indicators is one of the ways we track scale-up progress. … When I see the number of CHPs that are registered and the household enrollments increasing it means that scale-up progressing. We also look at reporting of key indicators on the dashboard which indicates that regular household visits are taking place. …It is now easier to do monthly reports to MoH’* (FGD2–3).

#### Infrastructure and interoperability

Infrastructure and interoperability are a key enabler of scale-up. Counties that already had infrastructure in place such as Kisumu, were the first to implement eCHIS, as it was more efficient to rely on what was already available. A senior ministry official reported that eCHIS is streamlined for interoperability with other HIS and its integration with existing systems at various levels is crucial especially to ensure that referrals are done with ease and that patient information flows easily from CHPs to health facilities.

*‘Mobile phone were provided by a key partner, Safaricom, … and in levels two, three, and four healthcare facilities, hardware like tablets and computers were provided along with the necessary software. Interoperability between eCHIS and other HIS was primarily managed through the health information exchange rather than direct integration with individual systems. The focus was on ensuring that data could be exchanged and accessed through a central client registry and shared health records. … Connectivity infrastructure was also established to support these implementations.’* (KII1).

A CHFP further emphasized the importance of device maintenance in ensuring that eCHIS usage was not impeded:

*‘If the mobile phone stops working, our CHPs can take it to the Safaricom customer care for repair. … The phones are also whitelisted so that the CHP does not use their data bundles…this is important to ensure that reporting is not interrupted.’* (FGD1–4).

#### Centralized management

The centralized national hosting of eCHIS offers distinct strategic advantages to the scale-up of eCHIS. It not only enhances oversight and management capabilities but also streamlines integration with other centrally deployed systems. Senior ministry officials articulated this as follows:

*‘eCHIS is accessed from our servers at MoH … all CHPs access the same standard forms for data entry. … Allowing each county to have their own versions of a community health system will mean that the information reported is different and decision making becomes difficult especially at the national level that provides resources.’* (KII1).

*‘On the factors that informed the scale-up of eCHIS, one other dimension we have primary care networks [PCN]. For these networks, the government is looking at a central digitalized system that links all the facilities, the different levels, that is including the community health levels. …the digital health platform includes eCHIS, and the other systems that are within the facilities so that they are linked. smartPCN will interlink the 314 sub-counties by a central system.… So as the government is looking at using the PCN model to implement UHC.’* (KII2).

### Barriers of eCHIS scale-up

Data gathered from KIIs and FGDs revealed six critical barriers to the scale-up of eCHIS. These barriers include: Challenges in securing adequate funding and resource limitations, logistical and infrastructure challenges, user management challenges, communication challenges and partner capacity shortfalls.

#### Challenges in securing adequate funding and resource limitations

The eCHIS implementation experienced resource constraints, primarily in funding and human resource such as in Embu County. This was because of the heavy reliance on donors and implementing partners, some of whom also faced resource shortages. This led to scale-up delays due to the unavailability of human and material resources for critical tasks like user training. Affected counties adopted a sequential approach to scale-up contingent upon available resources. A senior ministry official noted that:

*‘… we are mostly dependent on the partner and donor contributions to support the implementation of eCHIS. … Almost 95% are donor dependent. This has resulted in some delays where some donors fall short.’* (KII2).

An implementing partner further explained the role they played in supporting the process:

*‘Funding for eCHIS primarily came through grants, with collaboration between the division of community health and partners covering the initial 10 counties. Our involvement also extended to supporting to several counties, adding to the collaborative effort. The process involved significant resources for device acquisition, development, training, and implementation’.* (KII17).

In addition, a CHFP provided a detailed account of their deliberate choice to proceed with eCHIS scale-up sequentially, rather than through a simultaneous rollout:

*‘Our partner lacked the man-power to train all CHAs and CHPs at once and we therefore had to do it one subcounty at a time. … This meant that we also had to implement eCHIS one subcounty at a time.’* (FGD1–1).

#### Logistical and infrastructure challenges

Infrastructure and logistical challenges significantly influenced the pace and effectiveness of eCHIS scale-up across counties. Inadequate infrastructure, particularly server capacity, caused delays during the early implementation phase. For example, during the pilot in Kisumu County, the existing server infrastructure was insufficient to support a large number of users, resulting in the postponement of scheduled trainings and subsequent implementation activities until necessary upgrades were completed. In addition, logistical inefficiencies, such as delays in the delivery of critical resources including smartphones and training materials, hindered timely implementation in several counties, notably Migori, Kilifi and Tana River Counties. These issues were especially pronounced during the early stages of scale-up and in more remote counties that were also categorized as late implementers.

One CHFP noted the technical difficulties faced in the initial rollout of eCHIS:

*‘During the very first user training session, the servers could not handle the incoming traffic. … Training could not continue until this issue was dealt with in preparation for more users.’* (FGD2–1).

The provision of smartphones in some counties was inadequate, with the number of devices being fewer than the number of CHPs. A CHFPs reported that:

*‘In my county, some smartphones arrived early but they were not enough to kick start the training of the CHPs.’* (FGD2–5).

Another Explained that:

*‘On the training day, we had not received the smartphones and the training materials … we decided to have the training the following week. … not all the smartphones arrived on time.’*(FGD2–2).

A CHP explained that as a result of inadequate smartphones, eCHIS training was either postponed or limited to CHPs who had been issued a smartphone.

*‘When the first smartphones were sent to the county, not all of us received them. … and the county and partner decides to train some subcounties before the remaining phones arrived. … We did not receive all the phones we needed and some CHPs thought they could use their personal phones…’* (KII8)

Furthermore, a ministry official detailed the technical challenges encountered during the eCHIS initial rollout:

*‘One significant issue was the delay in the delivery of smartphones, which caused the project to fall behind the initial schedule. The phones were expected to be ready by June but were delayed due to the system and other logistical issues. Integration with existing health information systems also posed challenges. … However, these challenges were dealt with and did not significantly hinder the overall acceptance of the system.’* (KII4).

#### User management

User management presented several challenges, particularly due to the high attrition rates among CHPs after the initial training and difficulties in maintaining correct records. Key issues included scaling the system to support a larger user base and building the necessary infrastructure. Managing a large number of CHPs involved complexities like tracking stipends and keeping records up to date, highlighting the need for strong user management tools. A senior official from the ministry reported that:

*‘After training there was a large CHP attrition rate. Managing a large number of community health promoters involved complexities such as tracking stipends and maintaining accurate records. … Developing robust user management tools was crucial for addressing these challenges.’* (KII1).

#### Communication challenges

Communication with users during the initial eCHIS implementation and scale-up stages in Kisumu and Migori Counties encountered considerable challenges. A primary impediment was users’ general lack of awareness concerning the proper channels for reporting difficulties faced with the system. An implementing partner emphasized the need for effective communication strategies as follows:

*‘An area of improvement in eCHIS implementation activities to ensure efficiency is need for improved communication channels, particularly regarding notifying CHPs of the status of reported issues. It is important to have a standard tool for incidence reporting and information dissemination to improve response times. …However, aligning mindsets and making time to receive user feedback helped mitigate most challenges over time.’* (KII18).

#### Partnership capacity shortfalls:

This barrier to scale up was described from two perspectives. The first being the over reliance of partner organizations for funding and resources and the second being the lack of collaborations with partners who have the capacity to support the scale-up process. One CHFP stated that their scale-up approach was dictated by their partner’s ability to provide the necessary resources.

*‘Our county opted to implement eCHIS subcounty by subcounty, which is much slower … and was guided by the lack of funds to do a full scale implementation in the county. …Our implementing partner did not have the resources to do so.’ (KII17).*

A ministry official highlighted the importance of partnerships in the scale-up process as follows:

*‘Currently, eCHIS is implemented in 43 of the 47 counties across the country. One key lesson we have learnt is the importance of building strong partnerships. … The remaining counties, particularly in arid and marginalized regions, such as Garissa, Madera, Tana River, and Wajir still face challenges due to a lack of partner support. …they are expected to onboard by June 2024. All the counties beside Tana River are supported by partners. .... It has not yet implemented eCHIS … It is very important for us to collaborate.’* (KII1).

## DISCUSSION

The implementation of eCHIS or similar community-based HIS is underway in many African countries, including Ethiopia, Burkina Faso, Uganda and Zanzibar [31]. While early implementers like Ethiopia faced substantial challenges delaying full national scale-up [12], numerous countries have not yet achieved nationwide coverage. Many systems remain operational only in select districts or are undergoing pilot testing [12, 31]. This study documents Kenya’s nationwide eCHIS scale-up, identifying lessons learned by examining the approaches, enablers and barriers encountered.

Kenya’s eCHIS scale-up aimed for both horizontal reach, ensuring use by over 107 000 CHPs nationwide, and vertical depth through integration with existing systems and institutionalization within the healthcare framework. Achieving this required substantial funding, increased personnel and a formulated national community health implementation strategy [11, 30]. Kenya’s devolved healthcare system favoured both sequential and non-sequential scale-up approaches [11, 15], applied as follows:

A *sequential*, or phased, scale-up approach was predominantly utilized where resource mobilization was challenging [11, 34, 35], often due to the absence of a supporting partner or limited partner capacity for countywide scale-up. The national government adopted this approach because simultaneous nationwide implementation was impractical given its extensive resource demands and the complexities of Kenya’s devolved system. In the pilot county of Kisumu, sequential rollout facilitated iterative learning and system improvements. Similarly, counties like Migori and Embu implemented eCHIS progressively, scaling one subcounty at a time based on resource availability, such as smartphones and training personnel. Conversely, a *non-sequential*, or simultaneous, approach was employed in resource-rich counties [11, 34, 35] like Kakamega and Nakuru, where both the county and implementing partners had the capacity for immediate, full-scale rollouts.

The study revealed that context-specific enablers and barriers not only determined the chosen scale-up approach but also significantly shaped the eCHIS implementation timeline across counties. Consequently, counties could be categorized as pioneer, mid-phase, or late-phase implementers.

*Pioneer implementer* counties were characterized by strong county leadership and substantial partner support, providing the necessary resources for early initiation. Notable examples include Kisumu, Migori, Nakuru, Kilifi and Nairobi. *Mid-phase implementers*, while possessing some resources, adopted a more deliberative approach; their decision to implement eCHIS often depended on evidence of its performance and acceptance elsewhere. Examples include Embu, Bomet, Kakamega, Kirinyaga and Kiambu. *Late-phase implementer* counties typically had insufficient resources for early eCHIS implementation, often lacking an implementing partner. Furthermore, their unique cultural contexts, particularly in arid and marginalized regions, significantly influenced the implementation environment, as seen in Tana River and Marsabit counties. Understanding these characteristics can inform future strategies for HIS scale-up in countries with similar healthcare systems to Kenya’s.

Determinants of scale-up, both enablers and barriers, have been previously identified in studies on digital health intervention scale-up [12–15]. While this study corroborated these known factors, the following were particularly prominent enablers for Kenya’s eCHIS scale-up:

*Contextual Adaptation:* Tailoring eCHIS scale-up strategies to the devolved healthcare system and specific subnational conditions was essential. This involved flexible scaling, responsive to county-specific enablers and barriers, thereby promoting subnational autonomy in selecting appropriate approaches [13, 14].

*Strong Leadership and Government Support:* Vital national and subnational leadership facilitated effective eCHIS scale-up within the devolved system. The national government provided necessary support and political will, avoiding a rigid top-down approach that disregarded local contexts, thus empowering subnational governments [13, 15, 36].

*Partnerships and Collaborations*: These were key to the scale-up, as partners significantly supplemented governmental capacity, resources and reach. By offering financial support, resource mobilization, training, capacity building, technical expertise and implementation support, partners addressed critical gaps and accelerated progress [36, 37].

Despite inherent challenges, the eCHIS initiative delivered substantial results, primarily due to the effective mitigation of critical scale-up barriers and strong project management. This strategic approach was fundamental to achieving nationwide eCHIS implementation and its utilization by over 95% of CHPs. Leadership from the Division of Community Health and the Division of Digital Health was crucial in sustaining project momentum and strategic direction. Consequently, identified scale-up barriers were systematically managed:

To address limited partner capacity and strengthen financial support, the national government increased eCHIS budgetary allocations. Learning from the pilot phase, logistical impediments were mitigated by refining procurement and logistical frameworks, notably ensuring adequate delivery timelines. To overcome infrastructural weaknesses, the MoH enhanced system capacity for user traffic, while county governments partnered with Safaricom, the smartphone provider, for repair and maintenance services. User management improved through a dedicated tool ensuring accurate CHP records and facilitating correct stipend disbursement. Formal protocols for communication and addressing reporting challenges were also developed.

One notable remaining infrastructural challenge concerns smartphone loss by CHPs, which incurs penalties requiring device replacement by the CHP. The absence of an alternative phone for affected CHPs can subsequently preclude them from performing household visits, thereby impacting their operational duties.

The findings of this study provide valuable insights to inform HIS scale-up efforts in other LMICs with similar healthcare system characteristics, particularly those operating under devolved governance structures and facing persistent financial resource constraints. However, the applicability of these insights may be limited in contexts where healthcare systems are centrally managed or where financial constraints are not a significant barrier to HIS scale-up.

## CONCLUSION

The principle that ‘no one size fits all’ is central to understanding the success of the eCHIS scale-up documented in this study. The successful nationwide implementation and high utilization by CHPs depended on context adaptation and flexible scaling approaches. Crucially, strong national leadership provided vision and overarching support, empowering subnational governments to tailor scale-up approaches to local enablers and barriers. Furthermore, partnerships were pivotal in augmenting governmental capacity and addressing critical resource and expertise gaps. This study demonstrates that future HIS scale-up endeavors in diverse settings must prioritize tailored approaches, strong leadership and robust partnerships.

Looking ahead, future scale-up initiatives would benefit from a diversified funding portfolio to mitigate over-reliance on external partner organizations. This involves actively securing funding from multiple streams, including increased government budgetary allocations, continued engagement with donor organizations, and the exploration of viable public-private partnerships. Additionally, utilizing a Scale-Up Framework is recommended to provide structured implementation guidance, ensuring comprehensive preparation by focusing on leveraging potential enablers and proactively addressing or eliminating potential barriers [38].

This study offers valuable lessons for policymakers and implementers involved in HIS scale-up, informing future initiatives. A key recommendation is the development of comprehensive scale-up guidelines that delineate appropriate and effective approaches for different contexts, such as devolved systems of government in LMICs thereby facilitating successful HIS scale-up.

## Supplementary Material

eCHIS_Scale-Up_Questions_oqaf020

## Data Availability

The primary data from the eCHIS scale-up evaluation supporting this paper’s findings will be shared by the corresponding author upon reasonable request.
