# Supplementary material for: An exploration of the successful scale-up of the electronic community health information system in Kenya
Source: Oxf Open Digit Health. 2025 Sep 13;3:oqaf020. doi: 10.1093/oodh/oqaf020 (PMC12448183; doi:10.1093/oodh/oqaf020)
Supplement: eCHIS_Scale-Up_Questions_oqaf020 [file echis_scale-up_questions_oqaf020.pdf]

INTRODUCTION

Thank you for the opportunity to discuss with you.

My name is ______________________________, we are conducting a key informant interview as part of our ongoing system evaluation on the implementation of eCHIS in Kenya. This system evaluation activity is commissioned by the Division of Community Health and Supported by the University of Nairobi, HealthIT project.

Your expertise and insights are highly valued, and we appreciate your willingness to participate in this interview.

The purpose of this interview is to gather comprehensive information about the eCHIS implementation process, challenges faced, successes achieved, and potential areas for improvement. Your candid responses will greatly contribute to our understanding and help inform future strategies for enhancing community health services in Kenya. Your participation is entirely voluntary, and all information provided will be kept confidential. Thank you for your time and cooperation in advance.

Consent

I, ____________________________, voluntarily agree to participate in the key informant interview regarding the implementation of the eCHIS in Kenya. I understand that my participation is entirely voluntary, and I have the right to withdraw from the interview at any time without any consequences. I acknowledge that the information I provide will be used solely for system evaluation purposes and will be kept confidential. I also consent to the recording of the interview for accuracy in data collection and analysis.

I have been provided with contact information for any further questions or concerns regarding this interview.

Participant's Signature: _______________________________________ Date: ___________

Name (Optional):	
Gender (Optional):	
Occupation:	
Organization/position:	
Telephone number:	
E-mail address:	
In this interview, we will be discussing eCHIS scale-up	

Tool A - eCHIS Scale-up: National Level

KEY-INFORMANT INTERVIEW
eCHIS Scale-up Process at the National Level

NB: This interview is intended for individuals directly involved in the scale-up of eCHIS. This includes individuals from Divisions of Primary Healthcare and Community Health, Division of ICT, Division of Health Informatics, and Digital Health, and Implementing Partners.

eCHIS Background

1. Please tell us your role as a division/department/organization in eCHIS scale-up.

Pilot

2. What actions were taken in preparation for the piloting of eCHIS? (Prompt: Is there a checklist that was followed to ensure successful piloting?)
3. When did the eCHIS pilot begin and how long did it take?
4. In which counties was eCHIS piloted and why were they chosen? (Prompt: What was used to determine the suitability of a county for piloting?)
5. What lessons were learnt from the eCHIS pilot? (Prompt: What were the facilitators of implementation and the challenges that were faced during the pilot?)

After Pilot

6. How did you determine that eCHIS was ready to scale-up? (Probe: Is there a specific criteria it had to meet before being considered ready for implementation in other counties?)
7. When did the rollout of eCHIS to other counties begin?
8. Which counties were chosen for rollout once the pilot was complete? Why were they chose?
9. What are the actions that were taken in preparation for the rollout of eCHIS in the other counties? (Prompt: Is there a checklist that was followed to ensure effective scaling? Were any adaptations made to the system as it was scaled to the different counties?)
10. How many counties so far have implemented eCHIS? (Prompt: Are there counties experiencing more challenges than others with eCHIS implementation?)
11. What measures are in place to ensure the sustainability of the eCHIS in the country?

Scale-up Framework, scale-up approaches, enablers and barriers to scale-up

12. Which information systems scale-up framework was used to guide eCHIS scale-up and why? (Prompt: If no framework was used, what guided the scale-up process? and are there policies and strategies that guide the scaling-up of digital health technologies in Kenya?)
13. Is there an assessment tool in place to measure eCHIS scale-up? (Prompt: How is MoH monitoring scale-up and what factors are being tracked/measured to determine the success of scale-up?)
14. What approach(es) were used to scale up eCHIS? (Prompt: How was scale-up done? Were there different approaches for the national and county levels?)
15. What are the factors that enabled eCHIS to scale-up? (Prompt: What made scale-up seamless or successful?)
16. What challenges were encountered during eCHIS scale-up? (Prompt: What may have caused delays in implementation and scale-up – people, infrastructure, process, eCHIS, …?)
17. How did you address challenges during the scale-up process?

Recommendations and Lessons Learnt

18. In your opinion do you consider the nation-wide scale-up of eCHIS a success?
19. What lessons were learnt from the scale-up process that you would apply to future implementations or that you would recommend to other implementers looking to scale similar digital health interventions? (Prompt: Were the scale-up strategies effective or not? What would you suggest could have been added to the process, and what could been done differently?)

Is there anything else you would like to add?
Thank you for your valuable time.

Tool B - eCHIS Scale-up: County Level

KEY-INFORMANT INTERVIEW
eCHIS Scale-up Process at the County Level

NB: This interview is intended for individuals directly involved in the scale-up of eCHIS. This includes individuals from County Health Teams, and Implementing Partners.

eCHIS Background

1. Please tell us your role in eCHIS implementation and scale-up.
2. When did eCHIS implementation begin in your county and is it now fully implemented in the entire county?
3. What are the actions that were taken in your county in preparation for the rollout of eCHIS? (Prompt: Is there a checklist that was followed to ensure effective scaling? Were any adaptations made to the system to suit your county's needs)
4. What measures are in place to ensure the sustainability of the eCHIS within your county?

Scale-up Framework, scale-up approaches, enablers and barriers to scale-up

5. Which information systems scale-up framework was used to guide eCHIS scale-up in your and why? (Prompt: If no framework was used, what guided the scale-up process? Does your county have strategies and policies that guide the implementation and scaling-up of digital health interventions?)
6. Is there an assessment tool in place in your county to measure eCHIS scale-up? (Prompt: How is the county monitoring scale-up and what factors are being tracked/measured to determine the success of scale-up?)
7. Which scale-up approach did your county use? (Prompt: Was it a phased approach (one sub-county at a time) or a simultaneous approach (all sub-counties at once)?)
   a. What factors informed the choice of scale-up approach?
   b. If a phased approach was used, how many sub-counties so far have implemented eCHIS? (Prompt: Are the remaining sub-counties experiencing challenges with eCHIS implementation?)
8. What are the factors that enabled eCHIS to scale-up in your county? (Prompt: What made scale-up seamless or successful?)

9. What challenges were encountered during eCHIS scale-up in your county? (Prompt: What may have caused delays in implementation and scale-up – people, infrastructure, process, eCHIS,…?)
10. How did you address challenges during the scale-up process?

Recommendations and Lessons Learnt

11. In your opinion do you consider eCHIS implementation a success in your county? Why?
12. What lessons were learnt from the scale-up process that you would apply to future implementations or that you would recommend to other implementers looking to scale similar digital health interventions? (Prompt: Were the scale-up strategies effective or not? What would you suggest could have been added to the process, and what could been done differently?)

Is there anything else you would like to add?
Thank you for your valuable time.

Description of participants

**Tool**	**Key Informants**
**A - eCHIS Scale-up: National Level**	MoH –Those directly involved in the scale-up process from the Divisions of Primary and Community Health, Division of ICT, Division of Health Informatics, and Division of Digital Health, and Implementing Partners.
	Partners who may be able to answer on the piloting of eCHIS and scale-up from the national level.
**B - eCHIS Scale-up: County Level**	As many county focal persons as possible but at the least from:<br>county where eCHIS was piloted, county where scale-up took place one sub-county at a time, county where implementation took place in all sub-counties at one, county that implemented eCHIS late, county where eCHIS implementation has been very successful
	Partners who were involved at the county level
